# Abrupt Onset of Mutations in a Developmentally Regulated Gene during Terminal Differentiation of Post-Mitotic Photoreceptor Neurons in Mice

**DOI:** 10.1371/journal.pone.0108135

**Published:** 2014-09-29

**Authors:** Ivette M. Sandoval, Brandee A. Price, Alecia K. Gross, Fung Chan, Joshua D. Sammons, John H. Wilson, Theodore G. Wensel

**Affiliations:** 1 Verna and Marrs McLean Department of Biochemistry and Molecular Biology, Houston, Texas, United States of America; 2 Department of Molecular and Human Genetics, Baylor College of Medicine, Houston, Texas, United States of America; 3 Department of Vision Science, University of Alabama Birmingham, Birmingham, Alabama, United States of America; Universidade Federal do ABC, Brazil

## Abstract

For sensitive detection of rare gene repair events in terminally differentiated photoreceptors, we generated a knockin mouse model by replacing one mouse rhodopsin allele with a form of the human rhodopsin gene that causes a severe, early-onset form of retinitis pigmentosa. The human gene contains a premature stop codon at position 344 (Q344X), cDNA encoding the enhanced green fluorescent protein (EGFP) at its 3′ end, and a modified 5′ untranslated region to reduce translation rate so that the mutant protein does not induce retinal degeneration. Mutations that eliminate the stop codon express a human rhodopsin-EGFP fusion protein (hRho-GFP), which can be readily detected by fluorescence microscopy. Spontaneous mutations were observed at a frequency of about one per retina; in every case, they gave rise to single fluorescent rod cells, indicating that each mutation occurred during or after the last mitotic division. Additionally, the number of fluorescent rods did not increase with age, suggesting that the rhodopsin gene in mature rod cells is less sensitive to mutation than it is in developing rods. Thus, there is a brief developmental window, coinciding with the transcriptional activation of the rhodopsin locus, in which somatic mutations of the rhodopsin gene abruptly begin to appear.

## Introduction

Somatic mutations and other forms of genomic instability in post-mitotic neurons have been implicated in the development and progression of neural tumors and cancer, triplet repeat diseases, and other pathological conditions [1], [2], [3]. Deliberate manipulation of the cellular machinery involved in mutagenesis and DNA repair offers a way to edit the genome of neurons for experimental or therapeutic purposes [4]. Although extensive studies have been carried out on mutagenesis and DNA repair in cycling cells, especially in cell culture, little is known about such processes in post-mitotic neurons [5].

In order to study spontaneous mutations and cellular responses to DNA damage in rod photoreceptor cells of the mammalian retina, we have developed mouse models that allow highly sensitive detection of genetic changes at the rhodopsin (*Rho*) locus, which expresses the G-protein coupled receptor responsible for detection of photons of light in rod photoreceptor cells ([6], [7], [8]. Mutations in the rhodopsin gene are the most common cause of the retinal neurodegenerative disease, autosomal dominant retinitis pigmentosa (ADRP) [9]. Our studies are motivated by a desire to understand the fundamental mechanisms that regulate genomic stability and mutagenesis in these sensory neurons, and by the need to develop a sensitive test platform for gene-directed therapeutic interventions, including gene inactivation or gene correction in response to directed DNA damage.

We describe here the generation of a mutant version of the human rhodopsin-EGFP knockin mouse line [8], [10], designed to carry the ADRP-causing mutation Q344X, which introduces a premature stop codon and prevents GFP expression. Mice were engineered to express the mutant protein at low levels to minimize its toxic effects. Elimination of the stop codon allows full-length hRho-GFP expression, which can be easily identified by fluorescent microscopy, providing an exquisitely sensitive assay for detection of gene repair events in rod cells. By monitoring the number of green fluorescent rod cells in retinas, we can determine the frequency and timing of somatic mutations in the rhodopsin gene.

## Results

### Knockin human rhodopsin-EGFP mutant mice

**Figure 1:**
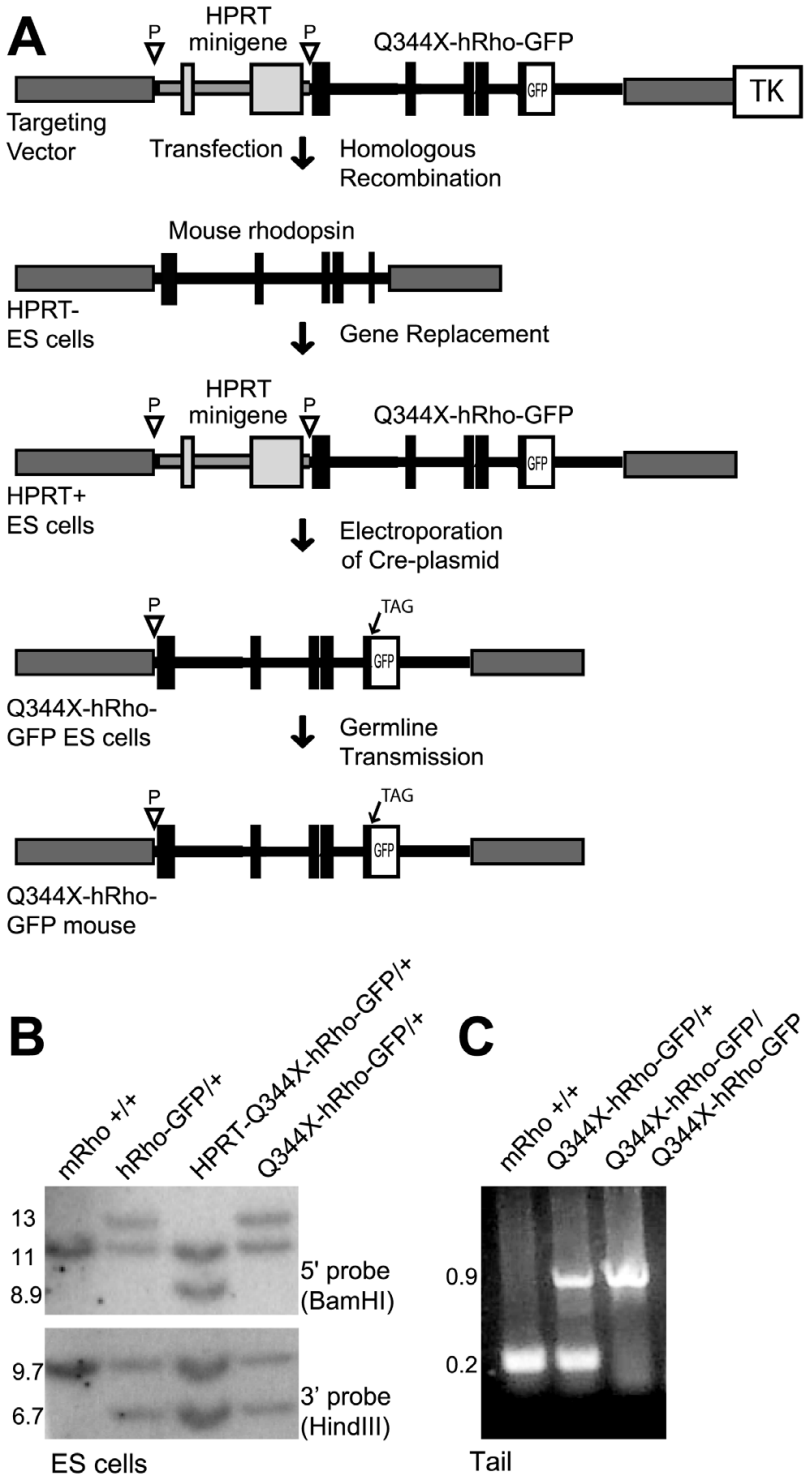
Mutant human rhodopsin-EGFP knockin mice. A. Diagram illustrating the procedure used to replace endogenous mouse rhodopsin with the Q344X-hRho-GFP gene. Gene replacement was accomplished by homologous recombination between the endogenous mouse rhodopsin locus and the homologous mouse sequences (dark grey) flanking the HPRT-Q344X-hRho-GFP gene on the targeting vector. Expression of Cre recombinase in HPRT-Q344X-hRho-GFP ES cells eliminated the HPRT minigene. Final mouse lines contain the Q344X-hRho-GFP fusion gene (shown in black, with exons as rectangles and introns as lines), preceded by a single loxP site (inverted triangle). B. Southern-blot analysis of ES cells. Restriction enzymes used to digest genomic DNA for analysis of the 5′- and 3′- ends of the modified locus are shown in parentheses, and fragment sizes are indicated in kilobases. The pattern of bands in hRhoG(H) heterozygous (hRho-GFP/+) mice served as a marker for the correct pattern of bands in Q344X-hRho-GFP heterozygous mice. C. PCR analysis of tail genomic DNA from heterozygous and homozygous Q344X-hRho-GFP mice.

We created a knockin mutant human rhodopsin-EGFP mouse line, Q344X-hRho-GFP, which carries a premature termination codon at amino acid position 344 (Figure 1A) [11], [12]. With the exception of the introduced C to T nonsense mutation, the rhodopsin gene is identical to one described previously for wild type human rhodopsin-EGFP mice (hRhoG(H)), hereafter referred to as hRho-GFP [8]. The gene encodes a mutant human rhodopsin protein linked to GFP at the C-terminus through the peptide APVAT. The human rhodopsin gene carries a loxP site in the 5′ UTR, which reduces rhodopsin expression about five-fold by interfering with mRNA translation [8]. This feature was introduced to diminish the potential deleterious effects of the dominant mutant proteins, providing an opportunity to test and analyze spontaneous somatic mutation in the healthy retinas of heterozygous mice.

### Rhodopsin expression

**Figure 2:**
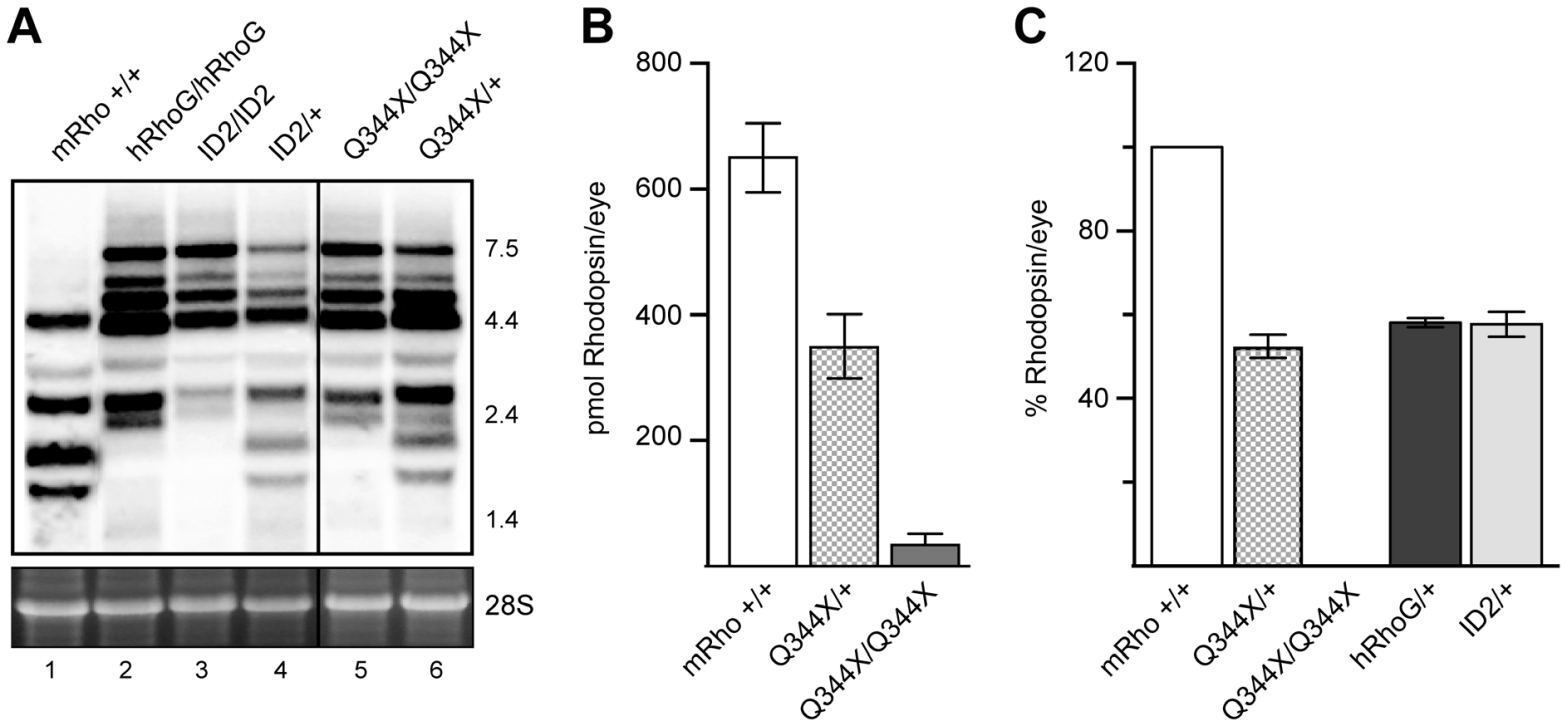
Rhodopsin transcript and protein levels in wild type and knockin mouse lines. A. Rhodopsin mRNA in wild type mice (mRho+/+), hRho-GFP homozygous mice (hRhoG/hRhoG), ID2-hRho-GFP homozygous (ID2/ID2) and heterozygous (ID2/+) mice, and Q344X-hRho-GFP homozygous (Q344X/Q344X) and heterozygous (Q344X/+) mice. Northern blot analysis used radiolabeled probes against human rhodopsin cDNA to detect human and mouse rhodopsin mRNA. Lane 1 shows the five species of mouse rhodopsin mRNA generated from the normal mouse rhodopsin locus. Lanes 2, 3, and 5 show the seven species of human rhodopsin mRNA generated from the knockin alleles. Lanes 4 and 6 show the mixture of species produced in heterozygous mice. Radioactive bands were quantified using a PhosphorImager and normalized for loading using the intensity of the 28S ribosomal rRNA bands in agarose gel before transfer (bottom). Marker sizes at the right of the gel are given in kilobases. B. Protein quantification by immunoblot of retina lysates probed with antibodies directed against the N-terminus of rhodopsin (mAb B630-N). Nine retinas were analyzed for each genotype. C. Total rhodopsin per retina as quantified by difference spectrophotometry. At least three retinas per genotype were analyzed.

To determine expression levels, we analyzed rhodopsin mRNA by Northern blot and rhodopsin protein by immunoblot analysis and difference spectrophotometry. We examined three different rhodopsin-EGFP knockin mouse lines: hRho-GFP, which encodes wild type rhodopsin fused to GFP [8]; Q344X-hRho-GFP; and ID2-hRho-GFP, which carries an internal duplication of exon 2 that generates at stop codon at position 178 [7]. As shown in Figure 2A, the intensities of the radiolabeled rhodopsin mRNA bands in the Northern blots were not significantly different. Thus, the levels of rhodopsin mRNA were not greatly affected by any of the rhodopsin mutations in these mouse lines.

We quantified protein levels by immunoblot analysis using B630-N antibody against the N-terminus of rhodopsin. Q344X-hRho-GFP heterozygotes had approximately half the total protein levels as their wild type littermates, which have two copies of the mouse rhodopsin gene. Retinas from Q344X-hRho-GFP homozygotes had much lower levels of rhodopsin (Figure 2B), in some cases about 5% of the amount of rhodopsin found in wildtype retinas, and in others too little to detect. We further confirmed these results by measuring the difference in absorbance at 500 nm between dark adapted and bleached samples, which provides a way to accurately calculate the amount of properly folded rhodopsin protein (Figure 2C) [13]. Reduced expression of the knockin allele is consistent with similar previous observations for wild type hRho-GFP mice[14] and for P23H-hRho-GFP mice [6], reflecting the presence of loxP site in the 5′ UTR of the gene, which impairs translation efficiency.

### Photoreceptor morphology and cell death

**Figure 3:**
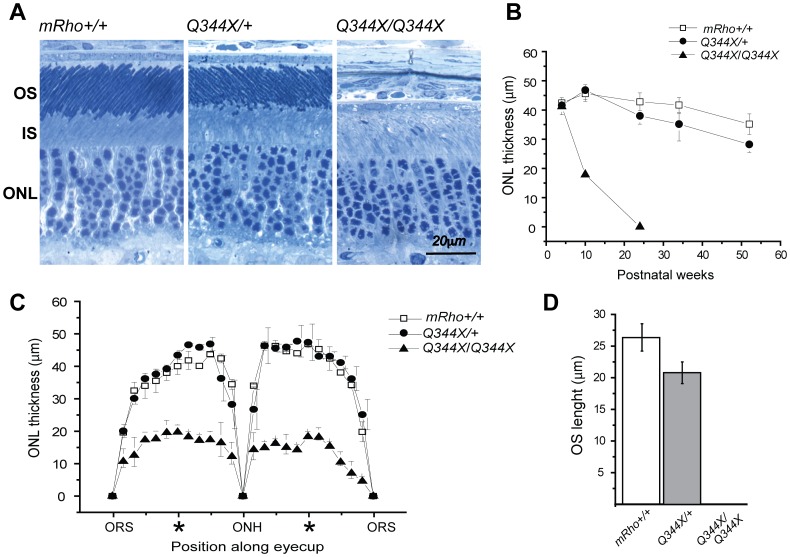
Morphological analysis of the photoreceptor cell layer in wild type and Q344X-hRho-GFP mice. A. Light micrographs of Epon resin embedded retinal sections of 4-wk-old littermates. The organization of outer segments (OS), inner segments (IS), and the outer nuclear layer (ONL) are shown for wild type mice (mRho+/+), heterozygous Q344x-hRho-GFP mice (Q344X/+), and homozygous Q344X-hRho-GFP mice (Q344x/Q344x). B. Loss of rod cell nuclei in the ONL with age. The thickness of the ONL in μm is plotted against age to show the rate of nuclear loss. Images were taken from the mid-eccentricity area indicated by * in C. C. Spidergrams of ONL thickness in retinas from wild type, Q344X-hRho-GFP heterozygous, and Q344X-hRho-GFP homozygous mice. We examined retinas from at least three different 10-wk-old mice for each genotype. Eyecup images were divided into 10 segments of equal size on each side of the optic nerve head (ONH) and extending to the ora serrata (ORS), which marks the end of the retina. Thickness of the ONL was measured over each segment. Each data point corresponds to an average of 10 measurements. D. Length of the outer segment layer in wild type, Q344X-hRho-GFP heterozygous, and Q344X-hRho-GFP homozygous mice. OS layer lengths (μm) were measured along the long axis of the outer segments in images from 4-wk-old mice retina sections.

To assess the effects of the knockin allele on the morphology and survival of rod photoreceptor cells, we analyzed retinal sections by light microscopy at different ages (Figure 3). The thickness of the outer nuclear layer (ONL) provides an estimate of the number of rods present at a given time. Q344X-hRho-GFP heterozygotes have a healthy retinal structure (Figure 3A), but lose about 33% of their rod cells over the course of 1 year (Figure 3B), consistent with the minimal degeneration observed in hRho-GFP mice [8], ID2-hRho-GFP mice [7], P23H-hRho-GFP mice [6], and mice heterozygous for a null mutation [15]. Q344X-hRho-GFP homozygous mice, on the other hand, showed rapid cell loss with complete degeneration by 24 weeks (Figure 3B). A spidergram analysis of sections collected at 10 weeks of age shows that cells die uniformly across the retina (Figure 3C). Notably, Q344X-hRho-GFP heterozygotes have rod outer segments that are about 20% shorter than their wild type littermates (Figure 3D), consistent with the notion that outer segments size depends on the amount of rhodopsin [15], [16], [17]. By contrast, Q344X-hRho-GFP homozygotes completely lack rod outer segments, even before any apparent cell death (Figure 3A), indicating that a normal C-terminus is essential for outer segment formation.

**Figure 4:**
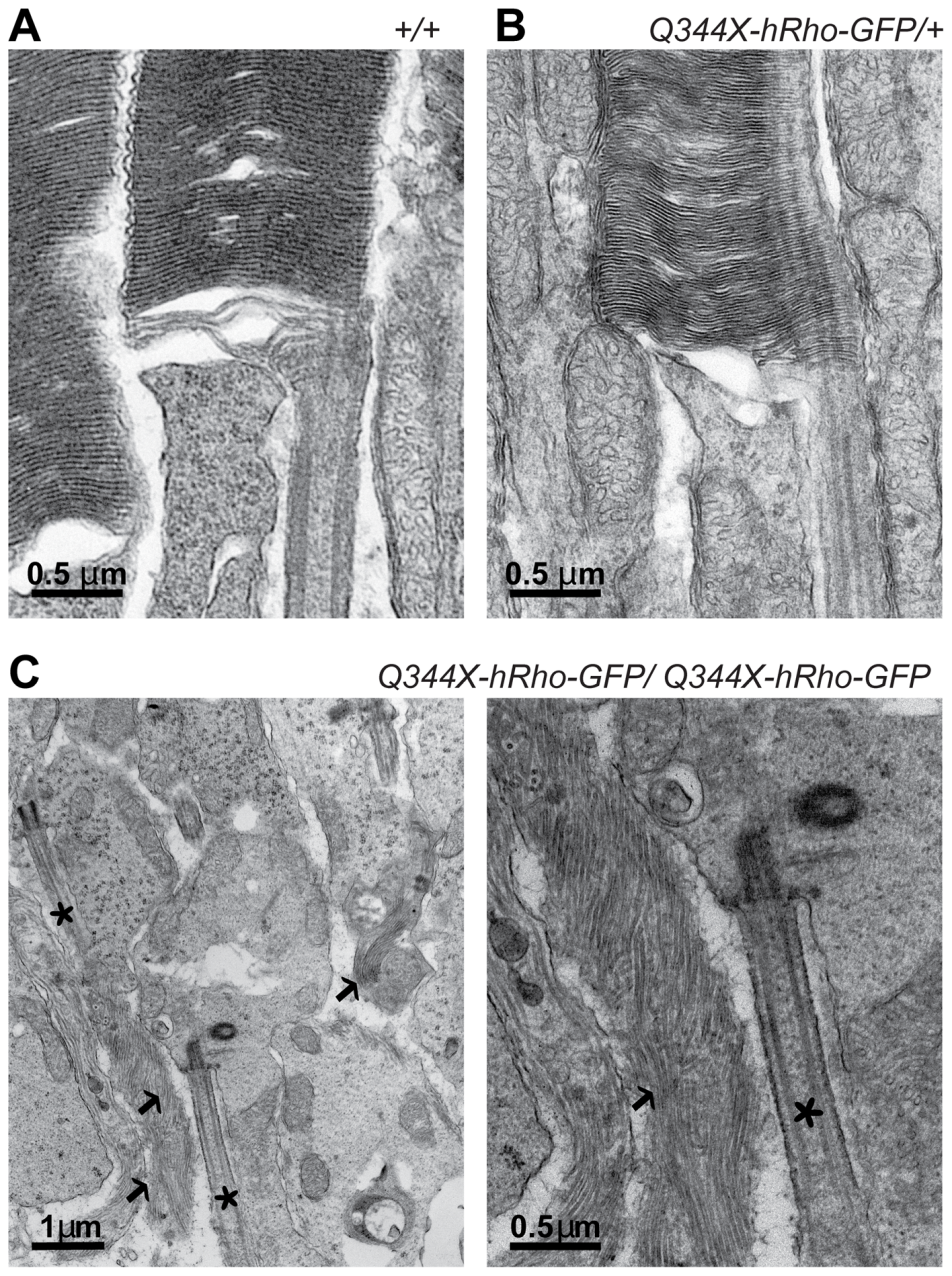
Transmission electron micrographs of rod photoreceptor cells in retinas of 4 week old mice. A. Structural organization at the base of the outer segment in wild type mice (+/+) B. Structural organization at the base of the outer segment in Q344X-hRho-GFP heterozygous mice (Q344X/+). In A and B, typical stacks of membranes disks are attached to the connecting cilium. C. Abnormal ultrastructure of rod cells in retinas from Q344X-hRho-GFP homozygous mouse (Q344X/Q344X). Isolated connecting cilia (asterisks) and disorganized membranes (indicated by arrows) are apparent, but no discernible rod-like structures are visible.

To characterize the morphology of photoreceptors in detail, we obtained high magnification electron microscopic images of Q344X-hRho-GFP retinas from one-month old mice (Figure 4). The ultrastructure of rod outer segments in retinas from heterozygous Q344X-hRho-GFP mice was indistinguishable from those in wild type retinas, with characteristic stacks of evenly spaced membrane disks, surrounded by plasma membrane and attached to the connecting cilium at the base of the outer segment (Figure 4A and 4B) [18] In contrast, rod cells from homozygous mice have isolated cilia and sparse clusters of disorganized membranes, with poorly defined cell boundaries (Figure 4C).

We conclude from these experiments that the retinas of Q344X-hRho-GFP/+ mice are sufficiently healthy to permit analysis of spontaneous mutation events.

### Spontaneous gene correction events

**Figure 5:**
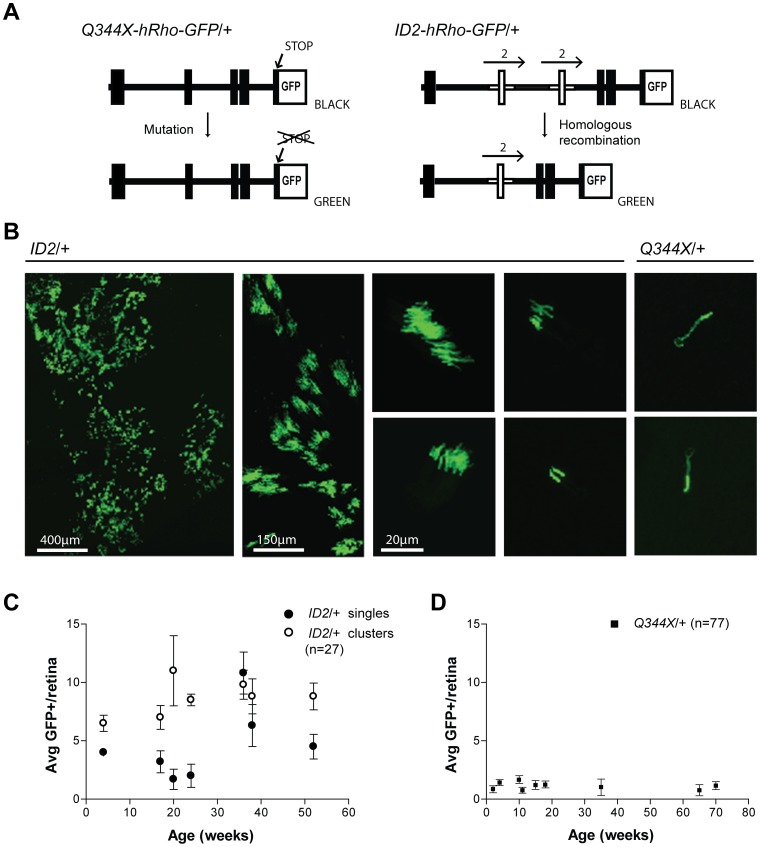
Spontaneous gene correction events in retinas of mutant mouse lines ID2-hRho-GFP/+ and Q344X-hRho-GFP/+. A. Schematic diagram of the structure of the ID2-hRho-GFP and Q344X-hRho-GFP genes and the mechanism leading to hRho-GFP expression. Exons are represented as black rectangles. In the ID2-hRho-GFP gene, the duplicated exon 2 is marked by horizontal arrows and shown in white. B. Projection of a stack of confocal images of a retinal wholemount, showing the GFP positive photoreceptors. The images of clusters of rod cells all came from ID2-hRho-GFP mice. The first image shows an extended supercluster of rod cells that was observed in a single mouse; the adjacent image shows an area of this retina at higher magnification. The next images show a variety of clusters arranged by decreasing number of rods per cluster The two images on the right are from Q344X-hRho-GFP retinas; they show individual rod photoreceptors that are overexposed to make the inner segments and nuclei visible. Scale bars are shown; the 20-μm scale bar applies to all the images without explicit scale bars. C. Age-dependent appearance of single green rods and clusters in ID2-hRho-GFP heterozygous mice. Retinas from several mice were examined at the indicated times and the values were averaged (age in weeks∶number of mice; 4∶2; 17∶6; 20∶3; 24∶2; 36∶4; 38∶4; 52∶6). Error bars indicate standard deviations. D. Age-dependent appearance of single green rods in Q344X-hRho-GFP heterozygous mice. Retinas were examined from several mice at the indicated times and the values were averaged (age in weeks∶number of mice; 2∶12; 4∶22; 10∶3; 11∶4; 15∶5; 18∶16; 35∶4; 65∶4; 70∶7). Error bars indicate standard deviations.

We used fluorescence and confocal microscopy to examine retinal whole mounts from Q344X-hRho-GFP/+ mice to quantify the frequency at which rods expressing GFP spontaneously arise. Because the GFP-tagged allele in Q344X-hRho-GFP mice contains a premature stop codon, GFP is not expressed in the vast majority of rod cells. However, any rod cell that undergoes a genetic change that eliminates the stop codon will express hRho-GFP, giving rise to bright green rods, which are readily visible in the otherwise nonfluorescent retina [7], [10]. The most common genetic change in these mice was expected to be a point mutation that converts the stop codon to an amino acid codon (Figure 5A). For comparison, we analyzed the retinas from ID2-hRho-GFP/+ mice, which we have described previously [7]. These mice carry a knockin rhodopsin gene with a duplicated segment that also introduces a premature stop codon. For ID2-hRho-GFP mice, however, we expected that homologous recombination between the duplicated segments would be the most likely way to eliminate the stop codon and allow expression of hRho-GFP (Figure 5A). These two lines of knockin mice allow us to examine spontaneous mutation and recombination events in rod photoreceptor cells.

In addition, the elegantly organized developmental process that gives rise to rod cells allows insight into the timing of mutation and recombination events. Because retinal progenitor cells divide to produce clusters of cells, the vast majority of which are rod photoreceptors [19], [20], genetic changes that occur prior to the last cell division will generate clusters containing two or more green rod cells, with earlier events giving rise to larger clusters. By contrast, genetic changes that occur in the last cell division or in terminally differentiated, non-dividing rod cells will give rise to single green rod cells. As shown in Figure 5B and summarized in Table 1, ID2-hRho-GFP mice produced individual green rod cells, as well as a variety of rod cell clusters containing from two to more than 30 green rod cells. In one remarkable instance, we observed a small patch of retina that contained more than 100 clusters. We presume that this supercluster arose by a recombination event in a very early retinal precursor.

**Table 1:** Analysis of spontaneous rhodopsin gene correction in mouse rod cells.

Mouse line	Total Events	Singles	Clusters	*n* a	Average^b^	Corrected^c^ average	Frequency^d^ (x 10^−7^)	Poisson^e^ s.d (x 10^−7^)	Sample^f^ s.d. (x 10^−7^)
Q344X-hRho-GFP/+	90	90	0	77	1.17	1.36	0.213	0.022	0.121
ID2-hRho-GFP	360	132	228	27	13.3	14.6	2.28	0.12	1.3

a *n* is the number of retinas analyzed.

b Average of spontaneous events per retina, which was calculated by dividing the total number of events by total number of retinas analyzed.

c The average number of events was corrected for age-dependent loss of rod cells, which occurs at a rate of 33% per year (Figure 3B) [7]. This correction assumes that this rate of loss applies equally to fluorescent and nonfluorescent rods. In Q344X-hRho-GFP/+ retinas, where only single green rods were observed, the correction is simple: at each age the number of green rods was fractionally increased to take into account the expected cell death. For ID2-hRho-GFP/+ mice, a similar correction for single rods was applied; however, in addition, clusters with more than 3 rod cells were assumed not to be affected and clusters with 2 rods were assumed to give rise to singles in an age-dependent manner.

d Frequency per rod cell, based on 64 million rods/retina[42] before decline.

e Assumes relative standard deviation of total counts, *xtot* used to derive frequency is *xtot* -1/2., and the relative standard deviation in the frequency is *xtot* -1/2/*xtot*.

f Calculated from actual sample variance as 
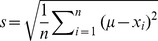
 where *n* is number of retinas scored, *μ* is the mean corrected number of events per retina, and x*_i_* is the corrected number of events in each retina *i*.

Counts of single green rods and clusters were determined in Q344x-hRho-GFP/+ and ID2-hRho-GFP/+ mice of several different ages, as shown in Figure 5C and D and summarized in Table 1. These data show three notable features. First, the frequencies of spontaneous events that give rise to green rods are very different in the two lines, with ID2-hRho-GFP mice experiencing about a tenfold higher frequency of events than Q344X-hRho-GFP mice. Second, the numbers of total events per retina do not increase substantially with the age of the mice; that is, the events do not appear to accumulate with time. Third, while both genotypes displayed single green rods, clusters of green rods were common only in ID2-hRho-GFP mice: they were not detected at all in retinas of Q344X-hRho-GFP mice. These observations place constraints on the timing of spontaneous mutation and recombination in rod cells in mice, as we discuss.

When we tested our counts of spontaneous events for age dependence, as described in the Materials and Methods, we found the result is, for *n* = 27 retinas, and 132 raw single events or 121 age corrected events, **μ** = 3.96. For the average at 4 weeks, 3.88, *p* = 0.16. For the average at 52 weeks, 2.57, *p* = 0.109. Thus, neither differs significantly from the population mean at the 90% confidence level. The result for Q344X is, for *n* = 77 retinas, and 90 raw single events or 104 age corrected events, **μ** = 1.36. For the combined average at 2 and 4 weeks, 1.24, *p* = 0.376. For average at 65 and 70 weeks, 1.70, *p* = 0.216. Thus, neither differs significantly from the population mean at the 80% confidence level.

## Discussion

We generated and characterized a mutant human rhodopsin-EGFP knockin mouse line–Q344X-hRho-GFP–in order to study mechanisms of mutagenesis and gene repair in terminally differentiated neurons. We had previously demonstrated that in the presence of a wild type mouse rhodopsin allele the hRho-GFP fusion protein localizes properly to the outer segment of photoreceptors, providing a quick and easy way to detect protein expression by fluorescence microscopy [8], [10]. However, in the Q344X-hRho-GFP gene construct, the nonsense mutation introduces an early termination codon at amino acid 344, preventing hRho-GFP expression and producing a truncated protein lacking its C-terminus sequence [11], [21]. Examination of retinas from heterozygotes confirmed that the majority of the cells are not fluorescent. Only in very rare occasions, at a frequency of 2.0×10^−8^ (Table 1), does a spontaneous mutagenic event allow expression of the hRho-GFP. These events were readily detected in retinal whole mounts as bright green fluorescent rod photoreceptors among millions of unmodified cells, demonstrating the exquisite sensitivity of the system (Figure 5).

In Q344X-hRho-GFP mice, spontaneous GFP expression could arise from single nucleotide mutations that change the TAG stop codon to an amino acid coding triplet or from small deletions that eliminate the stop codon and leave the transcript in frame. In mammalian cells, mice and yeast, single base substitutions tend to be one to two orders of magnitude more frequent than small insertion and deletions [22], [23], [24], [25]. Alternatively, mutations that introduce or activate a 3′ splice site between the stop codon and the GFP coding region would allow the stop codon to be skipped, giving rise to green rod cells. However, the small target–30 nucleotides between the stop codon and the start of the GFP gene–and its lack of resemblance to the consensus sequence for a 3′ splice site make this possibility unlikely [26]. In principle, mutations outside the rhodopsin gene could also suppress the stop codon or enhance readthrough to give green rods. Both these possibilities, however, would be expected to give lower levels of hRho-GFP expression, and the green rods from Q344X-hRho-GFP/+ mice are similar in intensity to those from ID2-hRho-GFP/+ mice.

Identifying the mutational changes that give rise to the green rods in Q344X-hRho-GFP/+ mice will require sequencing the human rhodopsin alleles in isolated cells. We have previously isolated green rod cells from the retinas of ID2-hRho-GFP/+ mice and analyzed their rhodopsin sequences [7]. Those studies, however, analyzed retinas in which 3% of the rods were green, due to ISceI-stimulated homologous recombination between the duplicated exons. Finding individual green rods in retinal preparations in which they are 10,000 to 100,000 times more rare is currently not feasible.

The lower frequencies of GFP positive cells in Q344X-hRho-GFP mice relative to ID2-hRho-GFP mice, in which GFP expression is possible only after elimination of the duplicated exon 2 by homologous recombination [7], indicates that the rate of spontaneous mutation is approximately tenfold lower than that of spontaneous recombination (Table 1). Similar ratios of point mutations to homologous recombination were observed at the APRT locus in Chinese hamster ovary cells [27], suggesting that the relative rates of these processes in dividing cells and differentiating neurons are comparable.

Unexpectedly, the frequencies of spontaneous reversion by mutation or homologous recombination did not increase substantially with time from 2 to 72 weeks of age in either Q344X-hRho-GFP or ID2-hRho-GFP mice (Figure 5C and D). The absence of an age-dependent increase in clusters of rods in Q344X-hRho-GFP mice is expected because they are generated only during rod cell development, when the retinal progenitor cells divide to produce clusters of rod cells, as well as other types of neurons that do not express rhodopsin [20] This developmental program provides insight into the timing of the recombination events that give rise to clusters of green rods. The number of green cells in a cluster indicates the time during cell proliferation when recombination occurred, with larger clusters corresponding to earlier events in development [19], [20]. The wide range of cluster sizes in ID2-hRho-GFP/+ mouse retinas indicates that recombination can occur at any time during this proliferative process (Figure 5B), as expected given the strong link between DNA replication and homologous recombination [28]. Even the single green rod cells in ID2-hRho-GFP retinas likely occurred during the last cell division.

In stark contrast, clusters of green rod cells are completely absent from the retinas of Q344X-hRho-GFP mice. This is a surprising observation. If the probability of mutation were constant during each cell division, we would expect roughly equal frequencies of clusters and single green rods, as is approximately true for the recombination events in ID2-hRho-GFP mice. The absence of clusters in Q344X-hRho-GFP mice indicates that all spontaneous mutations occurred in the last cell division or in cells that were no longer dividing. Studies of retinal development indicate that the cell divisions that give rise to terminally differentiated rod cells begin around embryonic day 15 and end around postnatal day 9 [29],[30]. The mutational process that gives rise to green rods in Q344X-hRho-GFP/+ mice must begin abruptly as proliferations ceases.

If mutation continued unabated at the same rate it began, with 1-2 green rods formed in the first two weeks of life, by the end of a year the typical retina should contain 25–50 green rods. Even accounting for the slow rate of cell death in these retinas (33% per year, Figure 3B), there should be substantially more green rods in older Q344X-hRho-GFP/+ mice than the 1–2 green rods observed. The lack of age dependence suggests two obvious possibilities. 1) The mutational process itself might be quickly switched off, producing a brief burst of green rods that persist with age. 2) The mutational process may continue as it began, but the green rod cells die as quickly as they are born, giving the illusion of stasis.

If the mutation rate is unchanged, the steady-state model requires that, on average, all the green cells born in one two-week period die in the next two-week period. We do not know what might cause rod cell death at such an extraordinarily high rate, one that is 10 times higher than the rate of rod cell death in homozygous Q344X-hRho-GFP mice (Figure 3B). It is highly unlikely that mutations in the rhodopsin gene could be so toxic, especially when expressed at the low levels characteristic of the engineered knockin locus. The heterozygous knockin rhodopsin alleles we have expressed at these low levels–nonmutant hRho-GFP [8], ID2-hRho-GFP [7], P23H-hRho-GFP [6], and Q344X-hRho-GFP–all cause retinal degeneration at the same slow rate as that observed in mice heterozygous for a null rhodopsin mutation, in which there is no observable decline in nuclei from 4 weeks to 6 months[17]. Thus, we consider mutation in the knockin rhodopsin gene to be a highly unlikely cause of rapid rod cell death.

It is also difficult to explain how mutations might arise in a brief burst in a defined, very narrow developmental window, which, as far as we know, is without precedent in neuronal differentiation. One notable event that occurs in this timeframe and might plausibly cause a mutational burst, is the activation of the rhodopsin gene, which is transcribed at a higher rate than any other gene in rod cells [31]. High rates of transcription have mutational consequences. In bacteria and yeast, transcription has been shown to increase spontaneous mutagenesis in a way that correlates with the rate of transcription [32], [33], [34]. Transcription-induced mutations arise predominantly on the nontranscribed strand, a pattern that is also evident in evolutionary comparisons of mammalian genomes and in the mutations that arise in rapidly dividing tumor cells [35], . During transit of RNA polymerase, the nontranscribed strand is periodically unpaired with its complement, becoming more susceptible to damage, which is thought to account for the observed mutational strand bias [34], [38].

In dividing cells, damage to transcribed genes is efficiently repaired by transcription-coupled nucleotide excision repair (TC-NER); however, TC-NER is strongly biased toward repair of the transcribed strand [39]. By contrast, in differentiated cells the nontranscribed strand is repaired equally as efficiently as the transcribed strand: a phenomenon termed differentiation-associated repair [40]. Thus, we speculate that the spike of mutations in the rhodopsin gene may be a consequence of the high rate of transcription of the rhodopsin gene in the transition period before the newly differentiated rod cells become fully capable of repairing damage to the nontranscribed strand. If this explanation is correct, our results identify what may be a general phenomenon associated with differentiation: newly transcribed genes in differentiating cells may experience a brief burst of mutations as the cells adapt to a new state of DNA repair.

Collectively, these results demonstrate that our knockin Q344X-hRho-GFP mouse model can report gene correction events in retina rod photoreceptors with very high sensitivity and good time resolution, providing an excellent system to probe mechanisms of DNA repair in photoreceptors cells during retinogenesis, as well as a powerful tool to test and optimize approaches aimed at editing the genomes of these cells as a means to treat degenerative diseases of the retina.

## Materials and Methods

### Animal Handling

Mice were handled in accordance with approved animal use protocols from the Institutional Animal Care and Use Committee at Baylor College of Medicine (Approval AN-1834) and in compliance with National Institutes of Health rules for the use of experimental animals. Euthanasia was carried out using CO_2_ inhalation for mature animals, and CO_2_ inhalation followed by decapitation of unconscious animals pups.

### Targeting the rhodopsin locus in mouse embryonic stem cells

Using the same homologous replacement strategy previously described [8], we substituted the mouse rhodopsin gene with one that encodes a mutant human rhodopsin-EGFP fusion protein. The targeting vector, which contains the sequence of a wild type human rhodopsin-EGFP fusion flanked by Lox sites, was modified by site-directed mutagenesis (QuikChange, Agilent Technologies). A single nucleotide substitution was introduced to replace the codon for glutamine (CAG) at amino acid position 344 with the TAG stop codon. The modified hRho-GFP segment was subcloned into a NotI site downstream of the HPRT minigene in a pBlueScript backbone. As shown in Figure 1A, the final targeting construct contains 5 elements: a floxed HPRT minigene and the herpex simplex virus TK gene, as selection markers; the mutant human rhodopsin-EGFP fusion gene (Q344X-hRho-GFP); and upstream (4.1 kb) and downstream (6.5 kb) flanking regions homologous to the mouse rhodopsin locus. Plasmid DNA was purified by gradient centrifugation in cesium chloride, linearized with BamHI restriction enzyme, and introduced in AB2.2 123 HPRT^–^ ES cells by electroporation. Potential knockin cells were selected for the presence of HPRT; random integrants were eliminated by selection against the hsv-TK gene. Positively targeted clones were identified by Southern blot analysis (Figure 1B).

### Q344X-hRho-GFP knockin mouse line

To eliminate the HPRT minigene from the targeted cells (Figure 1A), HPRT^+^ Q344X-hRho-GFP ES cells were electroporated with plasmid (pOG44), which expresses the Cre recombinase, and grown in 10 µM 6-thioguanine. Surviving HPRT^–^ cells were identified by Southern blotting and injected into blastocysts of C57Bl/6 Tyr mice (Figure 1B). Chimeric progeny were mated to C57Bl/6 mice and the pups were examined for germline transmission by PCR analysis of tail genomic DNA (Figure 1C). ES cell culture and blastocyst injection were performed by the Darwin Transgenic Mouse Core Facility at Baylor College of Medicine. Q344X-hRho-GFP mice were backcrossed ten generations to achieve a congenic C57Bl/6 strain background. Mice were handled in accordance with approved animal use protocols from the Institutional Animal Use and Care Committee at Baylor College of Medicine and in compliance with National Institutes of Health rules for the use of experimental animals.

### RNA quantification by Northern blot analysis

Northern analysis was performed as previously described [8]. Radiolabeled probes against human rhodopsin cDNA were used to detect human and mouse rhodopsin mRNA. Radioactive band intensity was measured using a PhosphorImager (Molecular Dynamics) and bands in different lanes were compared after normalization to a loading control: 28S ribosomal rRNA bands present in the agarose gel before transfer.

### Rhodopsin quantification by spectrophotometry and immunoblotting

Mice were dark-adapted overnight and euthanized with CO_2_ inhalation under dim red light conditions. Whole eyes were immediately extracted, frozen in liquid nitrogen, and saved at -80°C until ready to use. For spectrophotometry, each single eye was homogenized with rotor and pestle in 200 µL of ROS buffer [10 mM MOPS, pH 7.4; 30 mM NaCl; 60 mM KCl; 2 mM MgCl_2_; 1 mM dithiothreitol] supplemented with 1.5% w/v lauryldodecylamineoxide, 50 mM hydroxylamine, and proteinase inhibitor cocktail (Roche Diagnostics, Cat#11460400). The samples were spun down at 200 x *g* and the supernatant was used directly for spectrophotometry. Absorbance spectra were recorded at room temperature using an Olis-modified SLM-Aminco DW-2000 dual-beam instrument. Rhodopsin concentration was calculated by difference absorbance at 500 nm using an extinction coefficient of 42,700 M^−1^ cm^−1^.

For quantitative immunoblotting, retinas were homogenized in sample application buffer [2.5% (w/v) sodium dodecylsulfate, 25 mM Na_2_CO_3_, 7.5% (w/v) sucrose, 0.5% (v/v) beta-mercaptoethanol, 25 mM dithiothreitol], separated by standard polyacrylamide gel electrophoresis, transferred to a nitrocellulose supported membrane (Millipore, Inc.), blocked for 1 hour at room temperature with 4% non-fat dry milk in TBST [20 mM Tris-Cl, pH 7.6, and 0.1% (v/v) Tween 20], supplemented with 0.02% sodium azide, then incubated overnight at 4°C in the same solution containing primary antibody (monoclonal B630-N, hybridomas cell culture supernatant) [41]. Membranes were washed three times with TBST buffer and incubated with Goat anti-mouse IRDye800CW (Licor Biosciences) secondary antibody diluted 10^4^ in blocking buffer, washed with TBST buffer, and then imaged and analyzed with an Odyssey infrared imaging system.

### Light microscopy and transmission electron microscopy

After mice were humanely euthanized, eyeballs were cauterized on top to mark orientation, then enucleated and immediately fixed in ½ Karnovsky buffer [2% paraformaldehyde, 2.5% glutaraldehyde, 0.1 M cacodylate buffer pH 7.2] for 15 min at room temperature. The cornea, lens, and vitreous were removed and the eyecups were fixed overnight at 4°C in ½ Karnovsky buffer. The following morning tissue was washed twice with 0.1 M cacodylate buffer and post fixed in 1% osmium tetroxide in 1 N sodium phosphate buffer pH 7.2 for 2 hours. The tissue was rinsed with several changes of distilled water and dehydrated through a graded series of ethanol solutions. The tissue was progressively infiltrated with Epon resin and acetone mixture (1∶1 for 2 hours; 2∶1 for 3 hours; and 100% Epon overnight), and embedded in molds at 70°C for 8 hours. Eyes were sectioned transversally. One-micron thick and ultra thin 80-nm sections were obtained using a Leica Ultracut R microtome. One-micron sections were stained with toluidine blue and imaged using a Nikon Eclipse E400 light microscope equipped with a digital camera (DS-Fi1). Cell death was quantified by measuring the thickness of the ONL at different time points using NIS Elements image-analysis software, version 3.0, Nikon. Spidergrams were constructed by plotting ONL thickness (μm) as a function of position in the retina.

Ultra thin sections were cut and mounted on 100 mesh copper grids and stained with 2% uranyl acetate and Reynolds lead citrate. Grids were examined on a Zeiss CEM 902 electron microscope. Negatives were scanned with a Nikon Super Coolscan 9000 ED. All reagents were obtained from Electron Microscopy Sciences (Hatfield, PA USA).

### Retina whole mounts and confocal microscopy

Mice of different ages (4–70 weeks) were euthanized according to approved protocols. Eyeballs were collected and fixed in 4% paraformaldehyde in PBS for 45 min at room temperature and then washed in PBS. Cornea, lens, and vitreous were removed. The retina was carefully separated from the eyecup and mounted photoreceptor side up on a slide on a drop of VectaShield (Invitrogen) mounting media. The positions of GFP-positive cells were first determined in a wide-field fluorescence microscope, and then images of GFP positive cells were captured using a Leica TCS SP5 RS confocal microscope.

### Statistical treatment of spontaneous mutation and recombination

To test for any significant age-dependence of numbers of single spontaneous events, we carried out the following procedure. First, we corrected for the exponential decline in nuclei: for singles in ID2 heterozygotes, we subtracted the exponential decay of doubles into singles, and then normalized the result by the exponential loss of nuclei, using the measured rate constant of *k = 1/129.8 weeks* (loss of 33% at 52 weeks). After correction for decay of cells, the average number of starting doubles, *d_0_*, is 9.59. The corrected number of singles for each retina at age *t* weeks was calculated as:
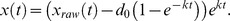



Next, we calculated the mean ***μ*** of the entire population of corrected frequencies of single events per retina ***μ*** *=* 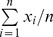, where *n = *27, the total number of retinas scored. Assuming a Poisson distribution, we calculated the probabilities of the average corrected counts per retina at age extremes *n*(4 weeks) and *n*(52 weeks), *i.e., p*(*n*(4weeks)) and *p*(*n*(52 weeks)) according to: 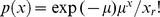 where *x_r_* is *x* rounded to the nearest integer.

The same treatment was applied to the single events counts for the Q344X retinas, except that there was no correction for decay of doubles, since none were observed, i.e., 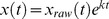, and the age extremes compared were the combined data for 12 retinas at 2 weeks and 22 retinas at 4 weeks, *vs.* the combined data for 4 retinas at 65 weeks and 7 retinas at 70 weeks.

## References

[1] Borgesius NZ, de Waard MC, van der Pluijm I, Omrani A, Zondag GC, et al (2011) Accelerated age-related cognitive decline and neurodegeneration, caused by deficient DNA repair. J Neurosci 31: 12543–12553. 21880916 10.1523/JNEUROSCI.1589-11.2011 PMC6703271

[2] Hoeijmakers JH (2001) Genome maintenance mechanisms for preventing cancer. Nature 411: 366–374. 11357144 10.1038/35077232

[3] Mollersen L, Rowe AD, Larsen E, Rognes T, Klungland A (2010) Continuous and periodic expansion of CAG repeats in Huntington's disease R6/1 mice. PLoS Genet 6: e1001242. 21170307 10.1371/journal.pgen.1001242 PMC3000365

[4] Palpant NJ, Dudzinski DM (2012) Zinc-finger nucleases: looking toward translation. Gene Ther. 10.1038/gt.2012.2 22318089

[5] Simonatto M, Latella L, Puri PL (2007) DNA damage and cellular differentiation: more questions than responses. J Cell Physiol 213: 642–648. 17894406 10.1002/jcp.21275

[6] Price BA, Sandoval IM, Chan F, Simons DL, Wu SM, et al (2011) Mislocalization and Degradation of Human P23H-Rhodopsin-GFP in a Knockin Mouse Model of Retinitis Pigmentosa. Invest Ophthalmol Vis Sci 52: 9728–9736. 22110080 10.1167/iovs.11-8654 PMC3341127

[7] Chan F, Hauswirth WW, Wensel TG, Wilson JH (2011) Efficient mutagenesis of the rhodopsin gene in rod photoreceptor neurons in mice. Nucleic Acids Res 39: 5955–5966. 21478169 10.1093/nar/gkr196 PMC3152346

[8] Chan F, Bradley A, Wensel TG, Wilson JH (2004) Knock-in human rhodopsin-GFP fusions as mouse models for human disease and targets for gene therapy. Proc Natl Acad Sci U S A 101: 9109–9114. 15184660 10.1073/pnas.0403149101 PMC428481

[9] Rossmiller B, Mao H, Lewin AS (2012) Gene therapy in animal models of autosomal dominant retinitis pigmentosa. Mol Vis 18: 2479–2496. 23077406 PMC3472929

[10] Wensel TG, Gross AK, Chan F, Sykoudis K, Wilson JH (2005) Rhodopsin-EGFP knock-ins for imaging quantal gene alterations. Vision Res 45: 3445–3453. 16139321 10.1016/j.visres.2005.07.016

[11] Sung CH, Makino C, Baylor D, Nathans J (1994) A rhodopsin gene mutation responsible for autosomal dominant retinitis pigmentosa results in a protein that is defective in localization to the photoreceptor outer segment. J Neurosci 14: 5818–5833. 7523628 10.1523/JNEUROSCI.14-10-05818.1994 PMC6576989

[12] Jacobson SG, Kemp CM, Cideciyan AV, Macke JP, Sung CH, et al (1994) Phenotypes of stop codon and splice site rhodopsin mutations causing retinitis pigmentosa. Invest Ophthalmol Vis Sci 35: 2521–2534. 8163341

[13] Hubbard R, Kropf A (1958) The Action of Light on Rhodopsin. Proc Natl Acad Sci U S A 44: 130–139. 16590155 10.1073/pnas.44.2.130 PMC335377

[14] Gross AK, Decker G, Chan F, Sandoval IM, Wilson JH, et al (2006) Defective development of photoreceptor membranes in a mouse model of recessive retinal degeneration. Vision Res 46: 4510–4518. 16979686 10.1016/j.visres.2006.07.012

[15] Liang Y, Fotiadis D, Maeda T, Maeda A, Modzelewska A, et al (2004) Rhodopsin signaling and organization in heterozygote rhodopsin knockout mice. J Biol Chem 279: 48189–48196. 15337746 10.1074/jbc.M408362200 PMC1351248

[16] Wen XH, Shen L, Brush RS, Michaud N, Al-Ubaidi MR, et al (2009) Overexpression of rhodopsin alters the structure and photoresponse of rod photoreceptors. Biophys J 96: 939–950. 19186132 10.1016/j.bpj.2008.10.016 PMC2716671

[17] Price BA, Sandoval IM, Chan F, Nichols R, Roman-Sanchez R, et al (2012) Rhodopsin gene expression determines rod outer segment size and rod cell resistance to a dominant-negative neurodegeneration mutant. PLoS ONE 7: e49889. 23185477 10.1371/journal.pone.0049889 PMC3503812

[18] Gilliam JC, Chang JT, Sandoval IM, Zhang Y, Li T, et al (2012) Three-dimensional architecture of the rod sensory cilium and its disruption in retinal neurodegeneration. Cell 151: 1029–1041. 23178122 10.1016/j.cell.2012.10.038 PMC3582337

[19] Turner DL, Cepko CL (1987) A common progenitor for neurons and glia persists in rat retina late in development. Nature 328: 131–136. 3600789 10.1038/328131a0

[20] Turner DL, Snyder EY, Cepko CL (1990) Lineage-independent determination of cell type in the embryonic mouse retina. Neuron 4: 833–845. 2163263 10.1016/0896-6273(90)90136-4

[21] Deretic D, Schmerl S, Hargrave PA, Arendt A, McDowell JH (1998) Regulation of sorting and post-Golgi trafficking of rhodopsin by its C-terminal sequence QVS(A)PA. Proc Natl Acad Sci U S A 95: 10620–10625. 9724753 10.1073/pnas.95.18.10620 PMC27944

[22] Lee AT, DeSimone C, Cerami A, Bucala R (1994) Comparative analysis of DNA mutations in lacI transgenic mice with age. FASEB J 8: 545–550. 8181674 10.1096/fasebj.8.8.8181674

[23] Hill KA, Buettner VL, Glickman BW, Sommer SS (1999) Spontaneous mutations in the Big Blue transgenic system are primarily mouse derived. Mutat Res 436: 11–19. 9878678 10.1016/s1383-5742(98)00024-6

[24] Zhang LH, Vrieling H, van Zeeland AA, Jenssen D (1992) Spectrum of spontaneously occurring mutations in the hprt gene of V79 Chinese hamster cells. J Mol Biol 223: 627–635. 1542110 10.1016/0022-2836(92)90979-t

[25] Lynch M, Sung W, Morris K, Coffey N, Landry CR, et al (2008) A genome-wide view of the spectrum of spontaneous mutations in yeast. Proc Natl Acad Sci U S A 105: 9272–9277. 18583475 10.1073/pnas.0803466105 PMC2453693

[26] Nakai K, Sakamoto H (1994) Construction of a novel database containing aberrant splicing mutations of mammalian genes. Gene 141: 171–177. 8163185 10.1016/0378-1119(94)90567-3

[27] Sargent RG, Brenneman MA, Wilson JH (1997) Repair of site-specific double-strand breaks in a mammalian chromosome by homologous and illegitimate recombination. Mol Cell Biol 17: 267–277. 8972207 10.1128/mcb.17.1.267 PMC231751

[28] Orii KE, Lee Y, Kondo N, McKinnon PJ (2006) Selective utilization of nonhomologous end-joining and homologous recombination DNA repair pathways during nervous system development. Proc Natl Acad Sci U S A 103: 10017–10022. 16777961 10.1073/pnas.0602436103 PMC1502498

[29] Rapaport DH, Wong LL, Wood ED, Yasumura D, LaVail MM (2004) Timing and topography of cell genesis in the rat retina. J Comp Neurol 474: 304–324. 15164429 10.1002/cne.20134

[30] Morrow EM, Belliveau MJ, Cepko CL (1998) Two phases of rod photoreceptor differentiation during rat retinal development. J Neurosci 18: 3738–3748. 9570804 10.1523/JNEUROSCI.18-10-03738.1998 PMC6793157

[31] Palczewski K (2012) Chemistry and biology of vision. J Biol Chem 287: 1612–1619. 22074921 10.1074/jbc.R111.301150 PMC3265841

[32] Reimers JM, Schmidt KH, Longacre A, Reschke DK, Wright BE (2004) Increased transcription rates correlate with increased reversion rates in leuB and argH Escherichia coli auxotrophs. Microbiology 150: 1457–1466. 15133107 10.1099/mic.0.26954-0

[33] Kim N, Abdulovic AL, Gealy R, Lippert MJ, Jinks-Robertson S (2007) Transcription-associated mutagenesis in yeast is directly proportional to the level of gene expression and influenced by the direction of DNA replication. DNA Repair (Amst) 6: 1285–1296. 17398168 10.1016/j.dnarep.2007.02.023 PMC2034516

[34] Kim N, Jinks-Robertson S (2012) Transcription as a source of genome instability. Nat Rev Genet 13: 204–214. 22330764 10.1038/nrg3152 PMC3376450

[35] Rubin AF, Green P (2009) Mutation patterns in cancer genomes. Proc Natl Acad Sci U S A 106: 21766–21770. 19995982 10.1073/pnas.0912499106 PMC2799788

[36] Majewski J (2003) Dependence of mutational asymmetry on gene-expression levels in the human genome. Am J Hum Genet 73: 688–692. 12881777 10.1086/378134 PMC1180696

[37] Green P, Ewing B, Miller W, Thomas PJ, Green ED (2003) Transcription-associated mutational asymmetry in mammalian evolution. Nat Genet 33: 514–517. 12612582 10.1038/ng1103

[38] Datta A, Jinks-Robertson S (1995) Association of increased spontaneous mutation rates with high levels of transcription in yeast. Science 268: 1616–1619. 7777859 10.1126/science.7777859

[39] Nouspikel T, Hanawalt PC (2000) Terminally differentiated human neurons repair transcribed genes but display attenuated global DNA repair and modulation of repair gene expression. Mol Cell Biol 20: 1562–1570. 10669734 10.1128/mcb.20.5.1562-1570.2000 PMC85340

[40] Nouspikel T, Hanawalt PC (2002) DNA repair in terminally differentiated cells. DNA Repair (Amst) 1: 59–75. 12509297 10.1016/s1568-7864(01)00005-2

[41] Adamus G, Zam ZS, Arendt A, Palczewski K, McDowell JH, et al (1991) Anti-rhodopsin monoclonal antibodies of defined specificity: characterization and application. Vision Res 31: 17–31. 2006550 10.1016/0042-6989(91)90069-h

[42] Jeon CJ, Strettoi E, Masland RH (1998) The major cell populations of the mouse retina. J Neurosci 18: 8936–8946. 9786999 10.1523/JNEUROSCI.18-21-08936.1998 PMC6793518

